# Precursor-surface interactions revealed during plasma-enhanced atomic layer deposition of metal oxide thin films by *in*-*situ* spectroscopic ellipsometry

**DOI:** 10.1038/s41598-020-66409-8

**Published:** 2020-06-25

**Authors:** Ufuk Kilic, Alyssa Mock, Derek Sekora, Simeon Gilbert, Shah Valloppilly, Giselle Melendez, Natale Ianno, Marjorie Langell, Eva Schubert, Mathias Schubert

**Affiliations:** 1 Department of Electrical and Computer Engineering, University of Nebraska-Lincoln, Lincoln, Nebraska 68588 USA; 2 Nebraska Center for Materials and Nanoscience, University of Nebraska-Lincoln, Lincoln, Nebraska 68588 USA; 3 Department of Physics and Astronomy, University of Nebraska-Lincoln, Lincoln, Nebraska USA; 4 Department of Chemical Engineering, Polytechnic University of Puerto Rico, San Juan, Puerto Rico; 5 Department of Chemistry, University of Nebraska - Lincoln, Lincoln, Nebraska 68588 USA; 6 Institutionen för fysik, kemi och biologi (IFM), Linköpings Universitet, SE-58183 Linköping, Sweden; 7 Leibniz Institut für Polymerforschung Dresden e.V., D-01005 Dresden, Germany

**Keywords:** Chemistry, Engineering, Materials science, Nanoscience and technology, Optics and photonics, Physics

## Abstract

We find that a five-phase (substrate, mixed native oxide and roughness interface layer, metal oxide thin film layer, surface ligand layer, ambient) model with two-dynamic (metal oxide thin film layer thickness and surface ligand layer void fraction) parameters (dynamic dual box model) is sufficient to explain *in-situ* spectroscopic ellipsometry data measured within and across multiple cycles during plasma-enhanced atomic layer deposition of metal oxide thin films. We demonstrate our dynamic dual box model for analysis of *in-situ* spectroscopic ellipsometry data in the photon energy range of 0.7–3.4 eV measured with time resolution of few seconds over large numbers of cycles during the growth of titanium oxide (TiO_2_) and tungsten oxide (WO_3_) thin films, as examples. We observe cyclic surface roughening with fast kinetics and subsequent roughness reduction with slow kinetics, upon cyclic exposure to precursor materials, leading to oscillations of the metal thin film thickness with small but positive growth per cycle. We explain the cyclic surface roughening by precursor-surface interactions leading to defect creation, and subsequent surface restructuring. Atomic force microscopic images before and after growth, x-ray photoelectron spectroscopy, and x-ray diffraction investigations confirm structural and chemical properties of our thin films. Our proposed dynamic dual box model may be generally applicable to monitor and control metal oxide growth in atomic layer deposition, and we include data for SiO_2_ and Al_2_O_3_ as further examples.

## Introduction

Transition metal oxides (TMOs) are subject of contemporary interest for many applications. A wide range of interesting electrical, optical, electrochromic, and photocatalytic properties make TMOs attractive for device applications^1–6^. TMOs are being exploited, for example, as efficient light absorber materials in photo-voltaic devices^7,8^, as ion-transport, and/or ion-storage materials in rechargeable batteries^9^, as active materials in switchable electrochromic optical windows^10–12^, in low-earth-orbit protective coatings for all-solid-state electrochromic surface heat radiation control devices^13,14^, in gas sensing devices^15,16^, and in photo-catalysis devices^6^. TMOs are often fabricated as thin films, where fabrication conditions critically influence the resulting thin film properties^17–19^. Various growth processes for the fabrication of TMO thin films have been developed by utilizing physical vapor deposition (PVD) such as magnetron sputtering^20–22^, thermal evaporation^23,24^, and chemical vapor deposition (CVD)^25–27^. Thin films deposited by PVD processes are often affected by thickness and composition non-uniformity. Adhesion failure and non-homogeneous coverage across highly-faceted surfaces are often reported^28–30^. CVD processes enable deposition of highly uniform thin films in the thickness range of nanometers to many micrometers^31–33^. However, CVD growth processes critically depend on reaction conditions such as temperature and flux gradients, and conform growth over non-flat surfaces can lead to anisotropic (direction dependent) growth rates.

**Figure 1:**
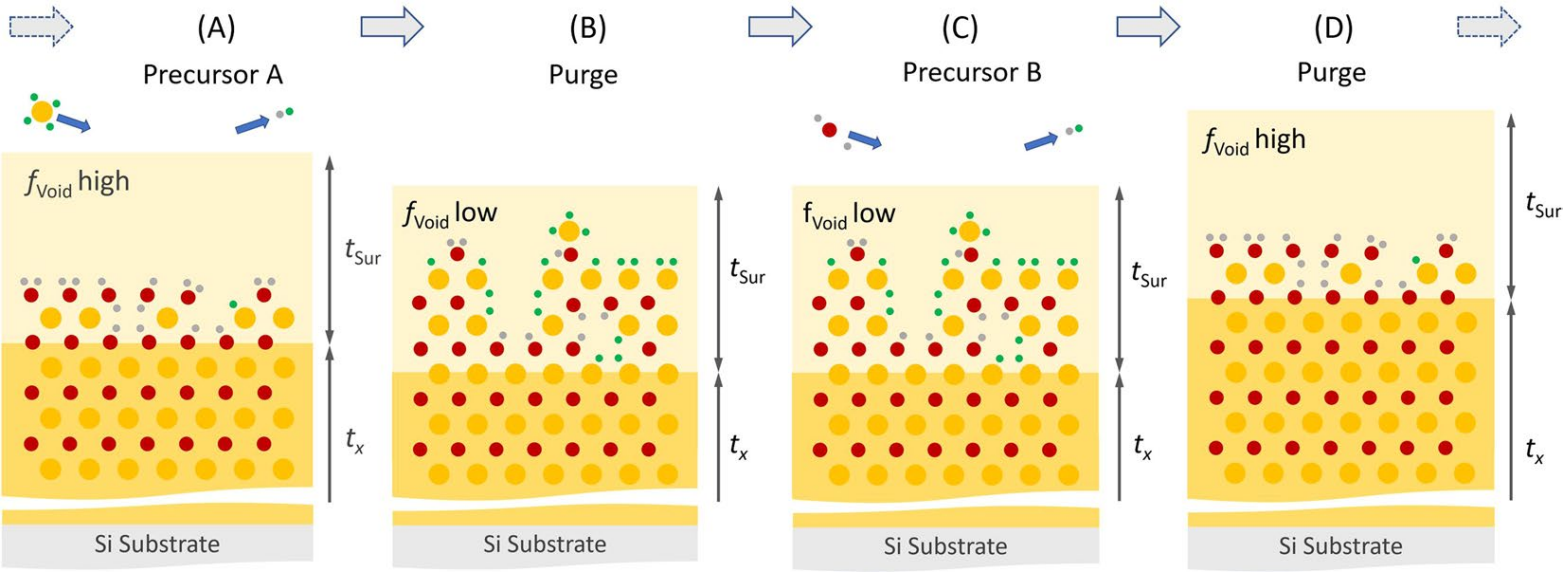
Schematic presentation of a dual precursor ALD process with surface roughness formation. (**A**) The first precursor is exposed to the surface which may already contain defects due to previous incomplete surface reactions. (**B**) Fast adsorption may lead to additional defect formation in the near surface region reducing the TMO layer thickness. (**C**) The second precursor reacts with the first precursor forming the desired metal oxide as well as converting the surface again to be susceptible for the first precursor. (**D**) A slow reaction kinetics for the second precursor can promote near surface defect reduction, leading to the positive net growth of the TMO layer. Non-reacted precursor materials are removed during periods with presence of inert purge gases (**B**,**D**). Two characteristic parameters, the TMO layer thickness (*t*_*x*_) and the void fraction (*f*_Void_) of a virtual surface layer with assumed thickness (${t}_{{\rm{Sur}}}=const.$), can be determined from analysis of *in-situ* spectroscopic ellipsometry (SE) data. The value of ${t}_{{\rm{Sur}}}=const.$ is determined from the native surface roughness of the substrate prior to deposition. See also Fig. 3.

Atomic layer deposition (ALD) is a CVD technique, which utilizes systematic and repeated introductions of gaseous-state precursors to a surface, while exploiting self-limited gas-solid reactions. ALD provides excellent control over layer-by-layer assembly of a desired material^34–36^. ALD is often employed when deposition of uniform and surface conform thin films are required^4,37–40^. Plasma-enhanced ALD (PEALD) permits deposition at lower substrate temperatures^41^. Physico-chemical conditions at surfaces determine reaction kinetics and processes, which lead to growth of TMO thin films (Fig. 1). TiO_2_ and WO_3_ have been widely reported in ALD growth^35,42–50^. Recipes involve the sequential use of a metal precursor (metal-halide, or metal-organic), a purging mechanism using an inert gas, and an oxygen source (often H_2_O, O_3_, or plasma excited O_2_)^51–53^. Use of plasma results in improved material properties such as high density as well as low-impurity content at lower deposition temperatures, while the growth per cycle is still comparable with non-plasma ALD processes^54^. Xie *et al*. compared precursors tetrakis(dimethylamido) titanium (TDMAT) and titanium tetraisopropoxide (TTIP) in combination with either water vapor, H_2_O plasma, or oxygen plasma^55^. Balasubramanyam *et al*. reported use of (tBuN)_2_(Me_2_N)_2_W and O_2_ plasma for growth of WO_3_ thin films^54^. H_2_O has been used routinely as a precursor in ALD, for example, for alumina and hafnium oxide^56,57^. Liu *et al*. employed H_2_O for WO_3_ ALD, and included a post-deposition oxygen annealing step reducing oxygen deficiencies^4^. Reinke, Kuzminykh, and Hoffmann studied the surface kinetics in ALD and determined reaction rate model parameters for TTIP and water^50^. It was found that the TTIP surface reaction is considerably faster than the hydrolytic reaction between water and adsorbed TTIP. Taking TiO_2_ as example, growth can occur by pyrolysis and/or hydrolysis reactions of the precursor molecules. Adsorbed TTIP species can either desorb or pyrolytically decompose on the substrate. Upon introduction of H_2_O^50^, or oxygen plasma^41^, hydrolytic reactions lead to the desired ALD growth. Purge periods in between precursor introduction phases remove unaffected precursor molecules. Hydrolysis is limited by available adsorbed precursor molecules and leads to the desired growth mechanisms, while pyrolysis is limited by the precursor flow leading to inhomogeneous growth. The balance between hydrolysis and pyrolysis is typically controlled by the substrate temperature. For TTIP and H_2_O, at 160 °C, the rate of hydrolysis is insufficient to completely hydrolyze the adsorbed TTIP molecules. At 260 °C, pyrolysis dominates the TTIP molecule decomposition. At 200 °C, the hydrolysis rate is increased leading to the reaction of a large fraction of adsorbed TTIP molecules with a total growth per cycle (GPC) of 0.46 Å^50^. A very similar GPC was reported by Potts *et al*. for PEALD at 200 °C using TTIP and oxygen plasma^41^.

Understanding of the complex processes that occur during the cyclic exposure of precursors on the surface is critically important to enable successful control of the growth. *In-situ* monitoring of the growth processes by fast and non-destructive physical methods can enable rapid development of new precursor materials and protocols, and can reduce efforts to find optimal growth parameters. To follow chemical surface reaction kinetics requires very fast monitoring capabilities. *In-situ* monitoring with resolution of few seconds cannot provide such information, but can permit real-time analysis of the thin film properties by observation of thickness and surface roughness evolution, for example. Well-known as non-destructive, non-contact, and fast optical characterization method, spectroscopic ellipsometry (SE) has been widely employed to study thin films and complex-layered heterostructures with thickness parameters ranging from fractions of Angstroms to several micrometers^38,46,58–63^. Klaus *et al*. suggested the application of *in-situ* SE during ALD growth processes^64^, and accurate thickness monitoring was reported for metal nitride thin films^41,65,66^ and metal oxide thin films^46,67–77^, for example. In these previous reports, *in-situ* SE data was measured once for every ALD cycle in order to determine the thickness GPC. Langereis *et al*. studied various metal oxide thin films, and determined thickness and optical constants also at half-cycles, after precursor introduction and surface stabilization^46^. Weber *et al*. studied hydrogenated amorphous carbon thin films, and implemented a parameterized model dielectric function approach. The authors reported deposition rate and discussed possible nucleation mechanisms for the thin film growth^78^. Kovalgin *et al*. determined electrical resistivity and thickness GPC from *in-situ* SE data model analysis measured in between cycles of hot-wire assisted ALD (HWALD)^75^. Rai and Argawal^79^ used *in-situ* attenuated total reflection Fourier transform infrared spectroscopy to study reaction mechanisms of TTIP and oxygen in PEALD of TiO${}_{2}$. Attempts to resolve the temporal evolution of the optical properties of the growing thin film during growth cycles in ALD using real-time *in-situ* SE have not been reported.

In this work, we report on the use of *in-situ* SE with few-second time resolution to investigate the evolution of TMO thin film properties during ALD growth processes. The time resolution is sufficient to monitor the changes of the optical properties of the growing surface in response to the changes of the growth parameters. SiO_2_, Al_2_O_3_, TiO_2_, and WO_3_ are chosen here as examples. In the Supplementary Material file, the results of both SiO_2_ and Al_2_O_3_ ALD processes are shown. We discuss a five-phase (substrate, mixed native oxide and roughness interface layer, metal oxide thin film layer, surface ligand layer, ambient) model with two-dynamic (metal oxide thin film layer thickness and surface ligand layer void fraction) parameters (dynamic dual box model). We use this model and explain the *in-situ* SE data measured within and in between multiple cycles. We measure *in-situ* SE data in the photon energy range of 0.7–3.4 eV with time resolution of approximately 2.5s. We discuss the observation of cyclic surface roughening and thickness GPC, and we suggest that processes due to precursor-surface interactions cause cyclic surface roughening and surface restructuring. We suggest use of our dual dynamic box model for unraveling surface modifications during atomic layer deposition processes leading to novel compounds such as Sb_2_Te_3_, perovskite SnTiO_3_, and potentially also for nitrides such as AlNi and TiN^80–82^.

## Results

ALD processes were performed as described in the Method section, and XPS, XRD, and AFM investigations (See Method section) confirmed structure, stoichiometry, and surface roughness after the ALD growth. Representative XRD, XPS, and AFM data are shown in the Method section. The TMO thin films are polycrystalline with random texture, and consist of orthorhombic (TiO_2_) and monoclinic (WO_3_) phases.

**Figure 2:**
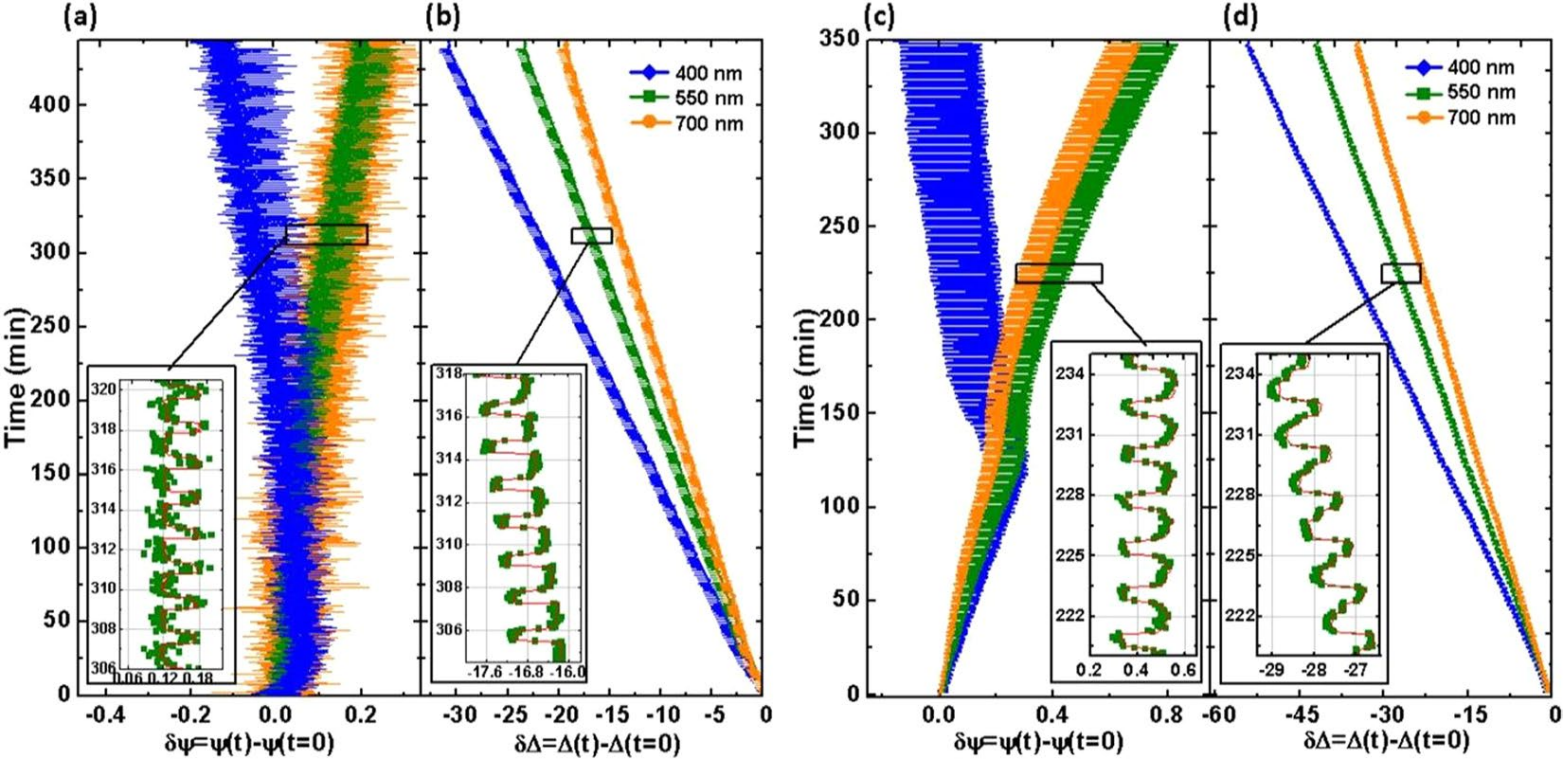
*In-situ* experimental (symbols) and best-match model calculated (red solid lines) ellipsometry data (*δ*Ψ: (**a**); *δ*Δ: (**b**)) of TiO_2_, and (*δ*Ψ: (**c**); *δ*Δ: (**d**)) WO_3_ thin films fabricated with 150 and 250 ALD cycles, respectively. Data are shown relative to Ψ and Δ values at the begin of the ALD processes, and for example at selected wavelengths of 400 nm (blue), 550 nm (green), and 700 nm (orange). The insets show examples of individual cycles for TiO_2_ and WO_3_ thin films.

Figure 2 shows the time (*t*) evolution of selected SE parameters Ψ (amplitude ratio of the p and s polarized components of the electromagnetic waves reflected from the sample surface) and Δ (phase difference between the p and s polarized components of the electromagnetic waves reflected from the sample surface), presented as changes relative to SE data measured prior to the ALD process start (*t* = 0); *δ*Ψ = Ψ(*t*) − Ψ(*t* = 0) and *δ*Δ = Δ(*t*) − Δ(*t* = 0). Data are shown for 250 cycles of TiO_2_ growth and 150 cycles of WO_3_ growth. Symbols depict the experimental data, solid lines correspond to the best-match model calculation using our dynamic dual box model. Data at three representative wavelengths out of all 588 measured wavelengths are shown. We note an excellent match between experiment and model calculation. We further note that similar match was obtained for all of the wavelengths investigated. SE parameters *δ*Ψ evolve linearly, except for cyclic modifications, while parameters *δ*Δ reveal variances with growth time, except for a similar cyclic behavior. It is noted that after reaching a certain time, parameters *δ*Δ diverge for different wavelengths, while parameters *δ*Ψ have each different slopes for different wavelengths regardless of time. We will explain this behavior below.

**Figure 3:**
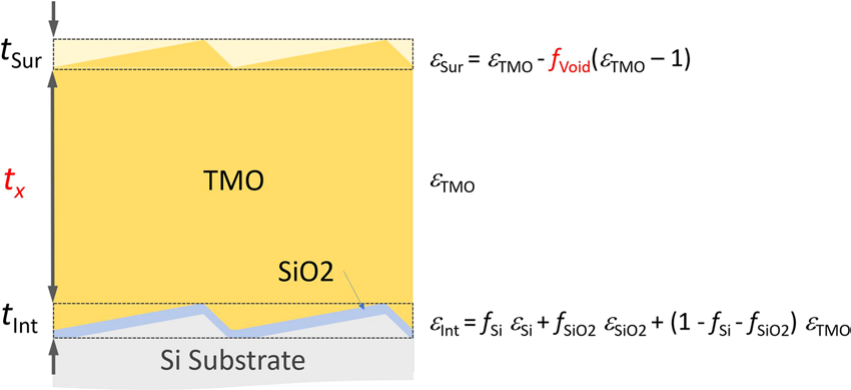
Schematic of the dynamic dual box model with the dynamic parameters (red): TMO thin film layer thickness (*t*_*x*_; *x* = ‘TiO_2_’, ‘WO_3_’) and surface ligand layer void fraction (*f*_Void_). The model consists of five phases (substrate, mixed native oxide and interface roughness layer with thickness *t*_Int_, TMO thin film layer with thickness *t*_x_, surface ligand layer with thickness *t*_Sur_, ambient). *t*_Sur_ is obtained from AFM measurements prior to and after the ALD growth. The dielectric functions of the three layers are calculated as indicated, with the dielectric functions of the substrate, the native oxide SiO_2_, and the TMO materials. The substrate is low-doped (100) oriented silicon.

In order to quantitatively analyze our recorded *in-situ* SE data, a physically meaningful model must be established. We present here a dynamic dual box model (Fig. 3). Within this five-phase (substrate, mixed native oxide and roughness interface layer, metal oxide thin film layer, surface ligand layer, ambient) model, two-dynamic (TMO thin film layer thickness and surface ligand layer void fraction) parameters are introduced in order to best-match model calculate the SE data measured during the ALD processes, from start to end. The substrate is low-doped (100) oriented silicon with dielectric function, ${\varepsilon }_{{\rm{Si}}}$. The dielectric function of the TMO thin film, is assumed constant during all growth steps, and determined in a different experiment as explained in the Methods section. The dielectric function of the surface ligand layer, ${\varepsilon }_{{\rm{Sur}}}$, is approximated by using a linear effective medium approximation (EMA), where parameter ${f}_{{\rm{Void}}}$ represents the unoccupied volume fraction within the top layer ${t}_{{\rm{Sur}}}$, and as explained in the Method section. The same EMA is used to calculate the dielectric functions for the mixed interface layer, ${\varepsilon }_{{\rm{Int}}}$, using the fractions and dielectric functions, ${f}_{{\rm{Si}}}$ and ${\varepsilon }_{{\rm{Si}}}$, and ${f}_{{{\rm{SiO}}}_{2}}$ and ${\varepsilon }_{{{\rm{SiO}}}_{2}}$, of the substrate and native oxide, respectively. The interface layer thickness, ${t}_{{\rm{Int}}}$, is approximated by the thickness of the SiO_2_ layer and the vertical surface roughness of the substrate, ${t}_{{\rm{Int}}}={t}_{{{\rm{SiO}}}_{2}}+{t}_{{\rm{Sur}}}$. The substrate surface roughness thickness parameter (${t}_{{\rm{Sur}}}$) and native oxide layer thickness parameter (${t}_{{\rm{Int}}}$) are assumed to remain constant during the ALD processes. The geometry of the growing surface is not flat to begin with due to the roughness of the substrate. We determine ${t}_{{\rm{Sur}}}$ from the arithmetic mean value of the vertical roughness of both the untreated substrate surface, and the as-grown TMO thin film surface obtained from analysis of atomic force microscopy (AFM) images. Thereby we define a virtual optical box of thickness ${t}_{{\rm{Sur}}}$ with effective dielectric function ${\varepsilon }_{{\rm{Sur}}}$. The equivalence of the surface roughness layer obtained from SE data analysis, and the surface roughness determined in AFM image analysis has been described previously^83,84^. Because this surface roughness layer is small compared to the wavelengths of the ellipsometric probe beam, the ultra-thin film limit is valid^85^. In the ultra-thin film limit, the thickness and the dielectric function of a layer cannot be differentiated during the model analysis of SE data. Instead, the SE data is very sensitive to the product of thickness and effective dielectric function only. Thus, if the thickness is known or if it can be ascertained from a reasonable argument then the effective dielectric function of such layer can be monitored very accurately. Because the effective dielectric function follows the linear EMA described by Rodenhausen and Schubert^85^, (see also Fig. 3), we thereby introduce the (volume) void fraction parameter, ${f}_{{\rm{Void}}}$. This parameter then reflects the unoccupied volume fraction of the virtual optical box of thickness ${t}_{{\rm{Sur}}}$. The portion of this layer containing TMO due to the substrate surface geometry defines the upper bound for ${f}_{{\rm{Void}}}$. Note that ${t}_{{\rm{Sur}}}$ is independent of the thickness of the TMO thin film layer, *t*_*x*_.

**Figure 4:**
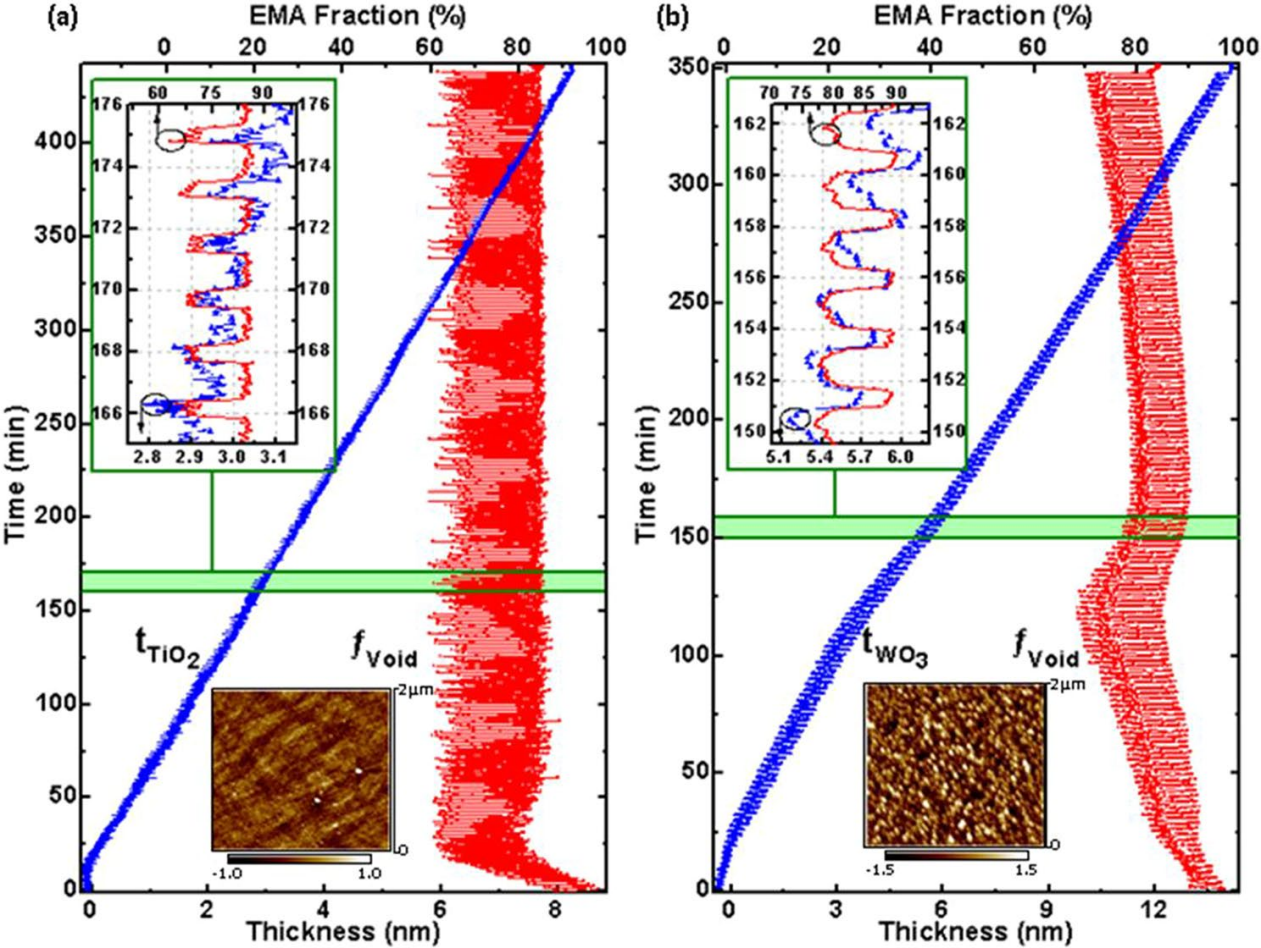
Best-match model calculated TMO thickness parameters (blue, triangle symbols) and surface ligand layer void fraction parameters (red, square symbols) obtained from the dynamic dual box model and the *in-situ* SE data shown in Fig. 2. The insets depict data sets during approximately 6 cycles. AFM images were taken at the end of the deposition process. The arithmetic mean value of the roughness depth parameters are obtained as 0.89 nm and 2.92 nm for TiO_2_ and WO_3_ thin films, respectively, and assumed as constant parameters *t*_Sur_.

The best-match model SE data shown in Fig. 2 as a function of time are obtained by only varying *t*_x_ (*x* = ‘TiO_2_’, ‘WO_3_’) and ${f}_{{\rm{Void}}}$. Figure 4 shows the resulting parameters ${t}_{{\rm{x}}}$ and ${f}_{{\rm{Void}}}$ as a function of time. Insets enlarge few cycle time periods, for better readability. Also shown in Fig. 4 are AFM images after deposition and exposure to normal ambient, and the average surface roughness, ${t}_{{\rm{Sur}}}$, was determined as 0.89 nm and 2.92 nm for TiO_2_ and WO_3_ thin films, respectively. Note that we have thereby reduced the time-dependent variations observed in Ψ and Δ at 588 wavelengths, i.e., the time-dependent evolution of 1,176 data points into the variations of two parameters versus time only. This noticeable reduction by obtaining a close match between experiment and model, seen in Fig. 2, is proof for the viability and correctness of our model to describe observed changes in the optical properties of TMO films during ALD processes. We note further that in our assumption, ${t}_{{\rm{Sur}}}$ is constant and we have implicitly assumed that the surface roughness due to the precursor-surface interactions does not reach the same order of magnitude than the topological surface roughness. If this assumption would be incorrect, i.e., if the precursor-surface interactions would lead to increasing surface roughness, parameter ${f}_{{\rm{Void}}}$ over many cycles would approach zero. However, as can be seen in Fig. 4, after initial growth steps, for both processes studied here, ${f}_{{\rm{Void}}}$ oscillates about a mean value with a large void fraction of ≈70–80%.

## Discussion

**Figure 5:**
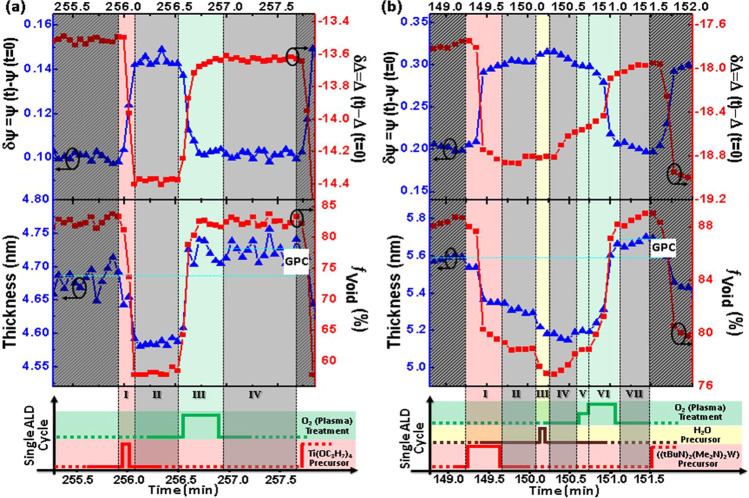
Evolution of the dynamic dual box model parameters during a single ALD cycle for (**a**) TiO_2_ and (**b**) WO_3_ [Experimental data: *δ*Ψ (red, squares); *δ*Δ (blue, triangles); $\lambda =550$ nm; best-match model calculated TMO thickness parameter *t*_*x*_ (blue, triangles); best-match model calculated surface layer void fraction parameter *f*_Void_ (red, squares)]. Overlaid are the ALD deposition phases, indicated by roman numerals (See also Tables 1 and 2 in Method section). Light-gray areas indicate phases without precursors present. Dark-gray areas to the left and right indicate neighboring cycles. Horizontal lines indicate the thickness GPC.

In Fig. 4, a nearly constant and linear evolution of parameter *t*_x_ is seen versus growth time for both processes. Both parameters, *t*_x_ and *f*_Sur_, reveal a stable, oscillatory behavior with almost constant magnitude, after a time of initial nucleation phase. The linear increase in *t*_x_ reflects a constant growth rate. The stability of the oscillations in *t*_x_ and *f*_Sur_ are best seen in the insets in Fig. 4. The variation in both parameters can be explained with dynamic processes occurring on the surface of the TMO thin films. A decrease in *f*_Void_ indicates reduced (incomplete) TMO surface coverage. If at the same time the TMO thickness parameter reduces this can be seen as indication for the propagation of surface defects (disorder) into the previously grown TMO thin film near-surface region, and which is shown as phases (B) and (C) in Fig. 1. Likewise, an increase in *t*_*x*_ with an increase in *f*_Void_ is indicative for a surface restructuring (reduction of roughness) and precursor removal from the surface while the TMO film is growing. If the processes driving these mechanisms are completely cyclic, i.e., not leading to a continuously increasing roughening of the surface, then a fully cyclic, i.e., recovering void fraction parameter will be seen. This is the case observed here for both processes. For the TiO_2_ process, *f*_Void_ is bound between approximately 58% and 83%, while for the WO_3_ process, the mean of *f*_Void_ is slightly varying over growth time, but oscillating by approximately 15%. Figure 5 depicts the evolution of the dynamic dual box model parameters during a single ALD cycle for both TMO thin films. The top panels depict a single-wavelength example of the measured SE parameters. The middle panel depicts the resulting parameters for TMO thickness and surface layer void fraction. The bottom row indicates the ALD phases and exposure time. A direct relationship is revealed between the ALD parameters and their time dependence with the observed dynamic dual box model parameters. The observed thickness and void fraction parameters can now be translated into precursor-surface interactions, and which repeat cyclically during the entire ALD process.

The TiO_2_ process is simpler as it only requires two precursors, oxygen and TTIP. During the introduction of TTIP within a very short time of 60 ms (phase I), a rapid modification of the thickness and void fraction is observed. Within about 3 data points, i.e., within approximately 15s, the ellipsometry instruments detects a reduction of the TMO thickness by approximately 17 Å, while the surface void fraction drops to its lowest value of approximately 58%. This behavior can be understood with a rapid attachment of precursor molecules and a disruption of the existing thin film surface. Such scenario is depicted schematically in Fig. 1(B). The process comes to an immediate halt after precursor gas removal in phase II. The surface remains stable during this phase. The incorporation of oxygen plasma over a period of 30s during phase III leads to recovery of the surface void parameter to approximately 83%, and an increase of the thickness by approximately 20 Å. This process can be understood by the reaction of the previous precursor to TiO_2_ and a surface reconstruction resulting in a net film thickness growth. This is indicated by the thickness GPC of approximately 0.34 Å in Fig. 5(a) at the end of phase IV. The total film thickness, which is obtained after 250 ALD cycles, is found as 8.35 nm. Therefore, the growth per cycle obtained from (total film thickness)/(total ALD cycles) is found as 0.334 Å/cycle in excellent agreement with the GPC found within each cycle. In the Supplementary Material, Table S-3 summarizes the GPC values for the SiO_2_, Al_2_O_3_, WO_3_, and TiO_2_ ultra thin films studied here with the same agreement noted. Such scenario is depicted schematically in Fig. 1(B). The process of forming TiO_2_ and restructuring the surface in phase IV is slower than the TTIP reaction with the surface in phase I. It is noteworthy to mention that the forming of the TiO_2_ and restructuring of the surface is self-limiting despite the continued presence of the second precursor during phase IV, and which is the hallmark of an ALD process. The surface remains stable after oxygen plasma removal until the begin of the next ALD cycle.

Similar precursor-surface interactions can be observed for the WO_3_ growth in Fig. 5(b). The sequence is more complex since three precursor materials are involved in the synthesis of this TMO. The introduction of (tBuN)_2_(Me_2_N)_2_W in phase I causes a similar reduction in TMO thickness and surface layer void fraction than observed for the TTIP introduction in the TiO_2_ process. The process is self-limiting and saturates during this phase. A continued surface restructuring is seen during the subsequent phase II of precursor removal via the continued decrease in void fraction. The introduction of the second precursor in phase III, H_2_O, causes further thickness and void reduction, indicative of continued surface restructuring and precursor surface hybridization. During the subsequent purging phase IV, the surface begins to react with the attached precursors, and after the introduction of hydrogen in phases V and VI, a rapid increase in thickness and a recovery of the void parameter is observed. The thickness increases by approximately 1 Å over the value at the begin of the cycle, establishing the thickness GPC for this process.

During the ALD process, the subsequent exposure of the surface to precursors, separated by purging agents leads to cyclic surface modifications, and the control of which may play a critical role in the resulting thin film quality. To the best of our knowledge, unraveling such complex processes have not been attempted previously by *in-situ* spectroscopic ellipsometry in ALD or PEALD growth of ultra-thin films^41,46,64–77^. The analysis of the evolution of the optical properties of ultra-thin films during growth cycles in ALD using *in-situ* SE as reported here in our work using the dynamic dual box model may gain further insight into the kinetics of the surface modifications within individual cycles. Thereby optically monitoring processes using *in-situ* SE may also help in faster identifying optimal growth recipes.

## Conclusion

*In-situ* SE permits the time-dependent observation of precursor-surface interactions in PEALD of TMO thin films, with the capability to resolve the evolution of layer thickness and surface roughness during separate steps of individual cycles. We introduced a five-phase model with two-dynamic parameters to analyse the ellipsometry data. The layer model is composed of substrate, mixed native oxide and roughness interface layer, TMO thin film layer, surface ligand layer, and ambient. Two dynamic parameters, the TMO thin film layer thickness and surface ligand layer void fraction, are sufficient to explain the *in-situ* SE data. The application of this model reveals cyclic surface roughening and thickness reduction with fast kinetics and subsequent roughness reduction and thickness increase with slow kinetics, upon cyclic exposure to precursor materials. We explain this observation by cyclic defect information and surface precursor interactions with restructuring and net film thickness growth. Structural, chemical, and surface investigations confirm the composure of our TMO thin films, and corroborate the findings observed from the *in-situ* SE analysis. We conclude that PEALD processes for TMO thin films of TiO_2_ and WO_3_ occur with subsequent surface roughening and restructuring, and which may be universal for TMO thin film growth. We further conclude that *in-situ* SE is a versatile tool which can be used to monitor precursor-surface interactions, and thereby reveal the processes, which can lead to net thickness growth and/or reduction.

## Methods

### Plasma-enhanced atomic layer deposition

PEALD of WO_3_ and TiO_2_ thin films was performed on silicon substrates using a Fiji F200 (Veeco CNT) instrument. The $(100)$ oriented wafers with native oxide were cut from low-doped, p-type conductive, B-doped, single crystalline silicon (University Wafers, (100) orientation). After sample insertion into the reactor, and prior to the main deposition processes, a 300 W oxygen plasma was applied for 300s in order to remove residual surface contaminants. Subsequently, a stabilization period was implemented to let the sample reach a steady state temperature. For TiO_2_, during each cycle the temperature of the Ti(OCH(CH_3_)_2_))_4_ (titanium tetraisopropoxide, TTIP) precursor was held at 80 °C while the temperature of the sample was maintained at 200 °C. At 200 °C, efficient hydrolysis leads to reaction of a large fraction of adsorbed TTIP molecules^50^. TiO_2_ was deposited using subsequent exposures of Ti(OC_3_H_7_)_4_ and a 300 W oxygen plasma to the sample surface with a vacuum purge between each exposure. The cycle parameters are listed in Table 1. For WO_3_ ALD, during each cycle the temperature of a (tBuN)_2_(Me_2_N)_2_W precursor was held at 80 °C while the temperature of the sample was maintained at 430 °C. WO_3_ was deposited using subsequent exposures of (tBuN)_2_(Me_2_N)_2_W, nanopure H_2_O (18.3 MΩ), and a 300 W oxygen plasma to the sample surface with a vacuum purge between each exposure. Pressurized argon was injected into the precursor cylinder in order to transport of the low volatility tungsten precursor to the sample reactor. The cycle parameters are listed in Table 2.

**Table 1:** TiO_2_ ALD deposition parameters.

STAGE	TTIP Pulse (s)	Oxygen Flow (sccm)	Oxygen plasma Pulse (s)	Argon Flow (sccm)	Argon plasma Flow (sccm)	chamber pressure (Torr)
I	*ON*(0.06*s*)	0	*OFF*	40	200	0.2
II	*OFF*	0	*OFF*	40	200	0.15
III	*OFF*	30	*ON*(30*s*)	40	200	0.6
IV	*OFF*	0	*OFF*	40	200	0.15

**Table 2:** WO_3_ ALD deposition parameters.

STAGE	(tBuN)_2_(Me_2_N)_2_W Pulse (s)	H_2_O Pulse (s)	Oxygen Flow (sccm)	Oxygen Flow Pulse (s)	Oxygen plasma Pulse (s)	Argon Pulse (sccm)	Argon plasma flow (sccm)	chamber pressure (Torr)
I	*ON*(3*s*)	*OFF*	0	*OFF*	*OFF*	60	260	0.49
II	*OFF*	*OFF*	0	*OFF*	*OFF*	60	260	0.37
III	*OFF*	*ON*(0.1*s*)	0	*OFF*	*OFF*	60	260	2.07
IV	*OFF*	*OFF*	0	*OFF*	*OFF*	60	260	1.87
V	*OFF*	*OFF*	50	*ON*(15*s*)	*OFF*	30	100	0.067
VI	*OFF*	*OFF*	50	*OFF*	*ON*(25*s*)	30	100	0.064
VII	*OFF*	*OFF*	0	*OFF*	*OFF*	60	260	0.37

### X-ray diffraction

**Figure 6:**
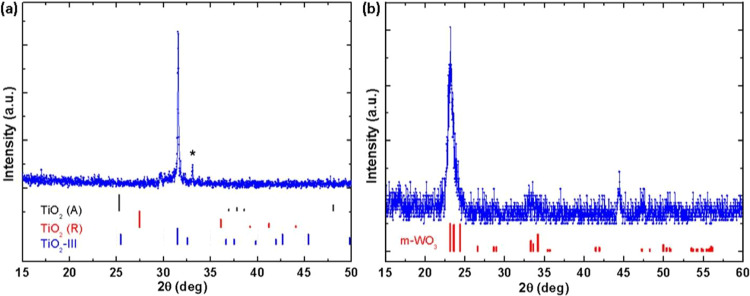
XRD patterns of (**a**) TiO_2_ and (b) WO_3_ thin films investigated in this work. Indicated are lattice plane positions for the orthorhombic (**a**) and for the monoclinic (**b**) phases of the polycrystalline TMO thin films. The black, red, and blue bar plots in (**a**) indicate the locations of calculated XRD peaks corresponding to the anatase, rutile and srilankate crystalline forms,respectively. The red bars in the part (**b**) indicates the calculated XRD peak locations for WO_3_ materials with monoclinic phase. The star symbol in the part (**a**) is the indicative of (100) oriented Si peak.

XRD data are shown in Fig. 6a,b for TiO_2_ and WO_3_, respectively. Measurements were performed with a Rigaku SmartLab Diffractometer using Cu-K_*α*_ radiation. Observed peaks in the TiO_2_ 2*θ* scan reveal a polycrsytalline thin film with orthorhombic phase (srilankite)^86^. For WO_3_, the diffracted intensities reveal a polycrystalline thin film with monoclinic phase^87^.

### Atomic force microscopy

AFM images were collected from all samples using a multi-mode atomic force microscope (Bruker-Nanoscope III). Image data were analyzed by using Nanoscope Visualization and Analysis software. The model surface roughness parameters of the investigated samples were calculated from the image data, and obtained as R_*q*_, the average of height deviation taken from the mean image data plane, and as R_*a*_, the arithmetic average of the absolute values of the surface height deviations measured from the mean geometric (flat) surface plane^88^. The average maximum profile height, derived from the average over all cutoff lengths (i.e., sampling lengths), the difference between the highest peak and lowest valley is denoted as R_*z*_. The corresponding R_*z*_ values are found as 0.89 nm and 2.92 nm for the TiO_2_ and WO_3_ thin films, respectively. We note that while the substrates for the two processes were taken from the same batch, the TiO_2_ process was conducted immediately after the package was opened, while the WO_3_ process was conducted 11 months later. Hence, we expect that the surface roughness for the WO_3_ is larger than for the TiO_2_ sample.

### X-ray photoelectron spectroscopy

**Figure 7:**
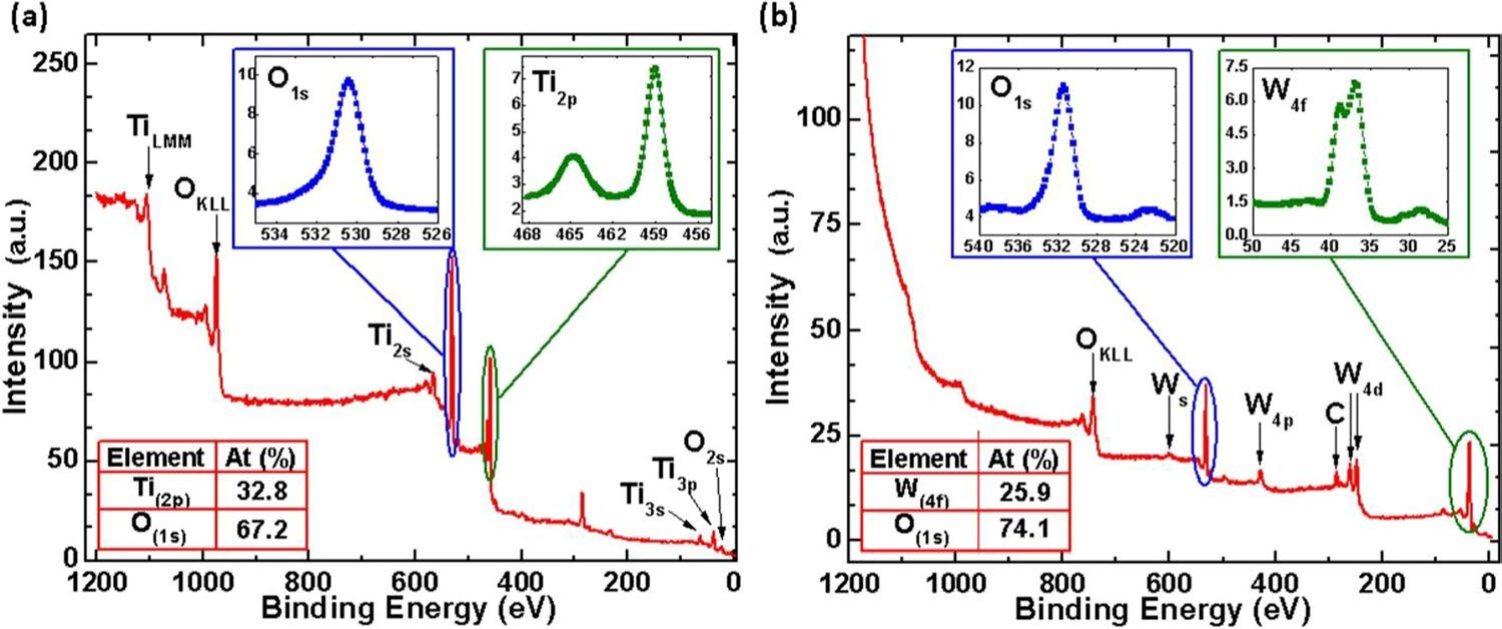
XPS spectra of the (**a**) TiO_2_ (**b**) WO_3_ PEALD thin films investigated in this work.

XPS spectra were acquired with a dual anode X-ray source and a hemispherical angle resolved electron analyzer (detector) inside an ultra-high vacuum (UHV) chamber at approximately 10^−10^ Torr. The X-ray source used the Mg-K*α* line at 1253.6 eV and data were taken at normal emission. The XPS data were analyzed utilizing the CASA software package^89^. The resulting XPS survey spectra corresponding to the samples fabricated with 250 ALD cycles (TiO_2_) and 150 ALD cycles (WO_3_) are presented in Fig. 7a,b, respectively. While the insets of Fig. 7a show the spectra for O(1s) and Ti(2p) core levels, the insets Fig. 7b show O(1s) and W(4f) core levels. We find that only oxygen and tungsten are present in the film, with a chemical composition of 74.1% and 25.9%, respectively. These values are in good agreement with what is expected for WO_3_ films.

### *In-situ* spectroscopic ellipsometry

*In-situ* SE data were measured with a multiple-wavelength (588 channels, 0.7–3.4 eV) ellipsometer with a rotating compensator instrument (M2000 FI, J.A. Woollam Co., Inc.). The ellipsometer was mounted to the ALD reactor at a fixed angle of incidence of 67.9°. Prior to data acquisition during the ALD processes the effects of vacuum port windows were determined and used during data analysis for proper reduction of optical window effects^58^. Data were recorded at approximately every 5s during WO_3_ ALD and at approximately every 3s during TiO_2_ ALD.

### Multi-sample data analysis

**Figure 8:**
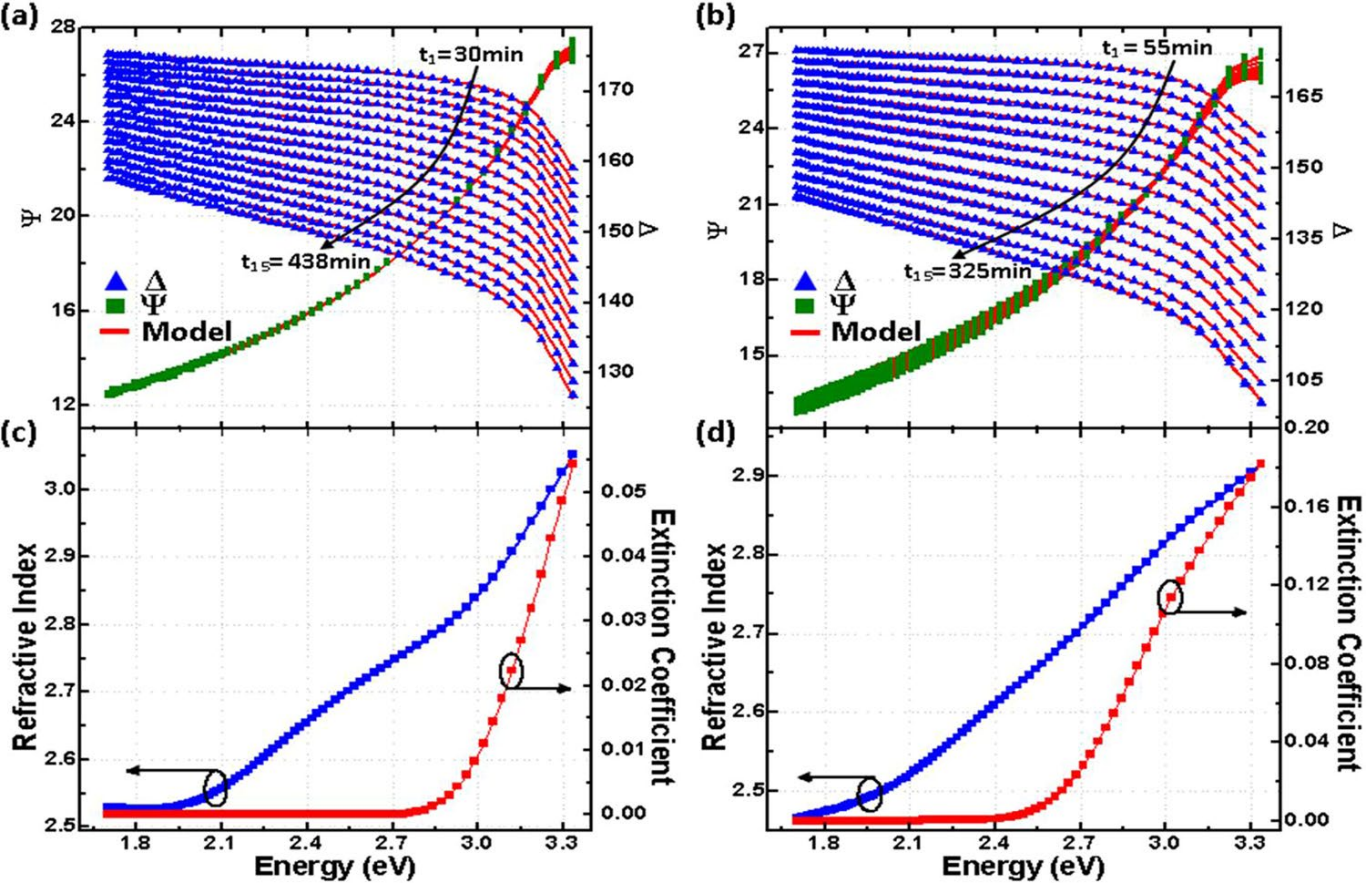
*In-situ* ellipsometry data $\psi$ (red symbols) and *δ* (blue symbols) at 15 equally spaced time slices from the data shown in Fig. 2, and best-match model results (red solid lines) for (**a**) TiO_2_ and (**b**) WO_3_ thin films. The best-match model calculated real (*n*, blue symbols) and imaginary parts (*k*, red symbols) of the complex refractive indices for TiO_2_ and WO_3_ are shown in (**c** and **d**), respectively.

Precise and accurate values for the TMO dielectric function, ${\varepsilon }_{{\rm{TMO}}}$, are performed retroactively after growth by analyzing selected data sets acquired during the growth. The analysis is based on the concept of the so-called multiple sample analysis. In this method, sets of SE data from sets of thin film samples with different thickness values but equal dielectric function are used^58^. If a given growth can be assumed to result in a homogeneous material thin film, then the assumption that the dielectric function of the thin film during growth is constant can be considered true. Then, sets of *in-situ* SE data at different growth times represent valid sets for a multiple sample analysis. SE data that were taken from the *in-situ* SE data selecting 15 equivalent cycle times spaced equally within 15 different time slices across the SE data set, and the dielectric functions in the spectral range of 0.71–3.45 eV were determined. Experimental and best-match model calculated SE data are shown in Fig. 8, together with the resulting best-match model calculated real (*n*) and imaginary (*k*) parts of the complex refractive indices for TiO_2_ and WO_3_. Spectra for *n* and *k* are then used to calculate the dielectric function, ${\varepsilon }_{{\rm{TMO}}}={(n+ik)}^{2}$, for the analysis of the time-dependence of the *in-situ* SE data and the dual dynamic box model in this work.

## Supplementary information

Supplementary Information.
