# Author Correction: Precursor-surface interactions revealed during plasma-enhanced atomic layer deposition of metal oxide thin films by* in-situ* spectroscopic ellipsometry

**DOI:** 10.1038/s41598-021-86624-1

**Published:** 2021-03-24

**Authors:** Ufuk Kilic, Alyssa Mock, Derek Sekora, Simeon Gilbert, Shah Valloppilly, Giselle Melendez, Natale Ianno, Marjorie Langell, Eva Schubert, Mathias Schubert

**Affiliations:** 1 Department of Electrical and Computer Engineering, University of Nebraska-Lincoln, Lincoln, NE 68588 USA; 2 Nebraska Center for Materials and Nanoscience, University of Nebraska-Lincoln, Lincoln, NE 68588 USA; 3 Department of Physics and Astronomy, University of Nebraska-Lincoln, Lincoln, NE USA; 4 Department of Chemical Engineering, Polytechnic University of Puerto Rico, San Juan, PR USA; 5 Department of Chemistry, University of Nebraska - Lincoln, Lincoln, NE 68588 USA; 6 Institutionen För Fysik, kemi och biologi (IFM), Linköpings Universitet, 58183 Linköping, Sweden; 7 Leibniz Institut Für Polymerforschung Dresden E.V., 01005 Dresden, Germany

Correction to:* Scientific Reports* 10.1038/s41598-020-66409-8, published online 25 June 2020

Giselle Melendez was omitted from the author list in the original version of this Article. This has now been corrected in the PDF and HTML versions of the Article, and in the accompanying Supplementary Information file.

The Author Contributions section now reads:

U.K. and D.S. performed ALD depositions of Al_2_O_3_, SiO_2_, TiO_2_, and WO_3_ thin films. U.K. took AFM images, collected in-situ SE data and designed the dynamic dual box model for in-situ SE data analysis. A.M. and G.M. contributed to the SE data analysis. S.V. and U.K. performed XRD analysis. S.G. and U.K. performed XPS analysis. N.I. and M.L. made contributions to the discussions over the experimental results. The manuscript was edited and approved by all authors. E.S. and M.S. supervised the project.

