# Supplementary material for: Precursor-surface interactions revealed during plasma-enhanced atomic layer deposition of metal oxide thin films by in-situ spectroscopic ellipsometry
Source: Sci Rep. 2020 Jun 25;10:10392. doi: 10.1038/s41598-020-66409-8 (PMC7316976; doi:10.1038/s41598-020-66409-8)
Supplement: Supplementary file 1 — Supplementary Information. [file 41598_2020_66409_MOESM1_ESM.pdf]

Supplementary material: Precursor-surface interactions revealed during plasma-enhanced atomic layer deposition of metal oxide thin films by *in-situ* spectroscopic ellipsometry

Ufuk Kilic[1,*], Alyssa Mock[1], Derek Sekora[1], Simeon Gilbert[3], Shah Valloppilly[2], Giselle Melendez[4], Natale Ianno[1], Marjorie Langell[5], Eva Schubert[1], Mathias Schubert[1,6,7,]**
[1]Department of Electrical and Computer Engineering, University of Nebraska-Lincoln, Lincoln, Nebraska, 68588, USA
[2]Nebraska Center for Materials and Nanoscience, University of Nebraska-Lincoln, Lincoln, Nebraska, 68588, USA
[3]Department of Physics and Astronomy, University of Nebraska-Lincoln, Lincoln, Nebraska, USA
[4]Department of Chemical Engineering, Polytechnic University of Puerto Rico, San Juan, Puerto Rico
[5]Department of Chemistry, University of Nebraska - Lincoln, Lincoln, Nebraska, 68588, USA
[6]Institutionen för fysik, kemi och biologi (IFM), Linköpings Universitet, SE-58183 Linköping, Sweden
[7]Leibniz Institut für Polymerforschung Dresden e.V., D-01005 Dresden, Germany
[*]ufuk.kilic@huskers.unl.edu, url:http://ellipsometry.unl.edu
[**]schubert@engr.unl.edu, url:http://ellipsometry.unl.edu

The oxygen-plasma-enhanced atomic layer depositions of SiO_2 and Al_2O_3 ultra thin films were successfully grown onto a (100) silicon substrates using a Fiji F200 (Veeco CNT) instrument and the time evolution of the thin film optical properties was measured by *in-situ* SE in the photon energy range of 0.7-3.4 eV. We applied our simple five-phase (substrate, mixed native oxide and roughness interface layer, metal oxide thin film layer, surfaceligand layer, ambient) model with two-dynamic (metal oxide thin film layer thickness and surface ligand layer void fraction)parameters (dynamic dual box model) and concluded that our model is capable of fully describing ALDs of both SiO_2 and Al_2O_3 TMO materials, as well.

In this supplementary material, we presented the resulting single cycle time evolution of the bulk thin film thickness parameter and the surface ligand layer void fraction together with the corresponding SE data, during each cycle, and throughout the entire deposition sequence. A s a conclusion, our model analysis permits determination of growth rate and identification of cyclic surface modifications during exposure to individual cycle steps for SiO_2 and Al_2O_3 TMO materials, as well. Our proposed dynamic dual box model may be generally applicable to monitor and control

metal oxide growth in atomic layer deposition.

In ALD process of SiO_2, $C_6H_{18}N_3Si$ (tris(dimethylamino) silane, TDMAS) precursor was employed as the main precursor. The temperature of the sample was maintained at 250°C. SiO_2 was deposited using subsequent exposures of $C_6H_{18}N_3Si$ and a 300 W oxygen plasma to the sample surface with a vacuum purge between each exposure. The cycle parameters are listed in Tab. S1.

Table S1 SiO_2 ALD deposition parameters.

STAGE	TDMAS Pulse (s)	Oxygen Flow (sccm)	Oxygen plasma Pulse (s)	Argon Flow (sccm)	Argon plasma Flow (sccm)	chamber pressure (Torr)
I	$ON(0.4s)$	0	OFF	30	100	0.19
II	OFF	0	OFF	30	100	0.1
III	OFF	50	$ON(20s)$	30	100	0.6
IV	OFF	0	OFF	30	100	0.1

For Al_2O_3, $Al(CH_3)_3$ trimethyl-aluminum (TMA, Strem 98%) precursor was employed as the main precursor. The temperature of the sample was maintained at 150°C. Al_2O_3 was deposited using subsequent exposures of $Al(CH_3)_3$ and a 300 W oxygen plasma to the sample surface with a vacuum purge between each exposure. The cycle parameters are listed in Tab. S2.

Table S2 Al_2O_3 ALD deposition parameters.

STAGE	TMA Pulse (s)	Oxygen Flow (sccm)	Oxygen plasma Pulse (s)	Argon Flow (sccm)	Argon plasma Flow (sccm)	chamber pressure (Torr)
I	$ON(0.06s)$	0	OFF	60	200	0.25
II	OFF	0	OFF	60	200	0.17
III	OFF	50	$ON(15s)$	30	100	0.6
IV	OFF	0	OFF	30	100	0.1

Table S3 Obtaining the growth per cycle (GPC) which are (first row) measured by an arbitrarily chosen single cycle and (second row) overall total thickness divided by number of cycles for Al_2O_3, SiO_2, TiO_2, and WO_3. The bottom two rows list total thickness(t_{Total}) and number of cycles (n).

	Al_2O_3	SiO_2	TiO_2	WO_3
$GPC_{Single\ Cycle}$ (nm)	0.88	0.53	0.34	0.91
$GPC_{Overall}=t_{Total}/n$ (nm)	0.89	0.54	0.334	0.9
t_{Total} (nm)	4.0	4.1	8.35	13.48
Number of cycles	45	75	250	150

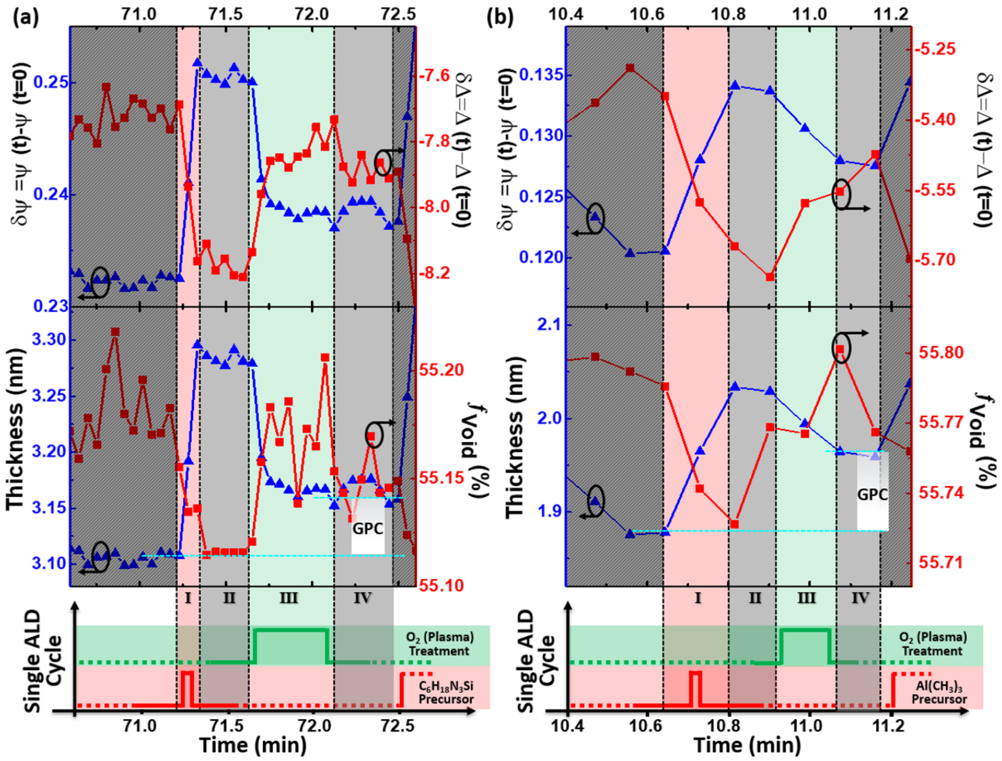

Fig S1 Evolution of the dynamic dual box model parameters during a single ALD cycle for (a) SiO_2 and (b) Al_2O_3 [Experimental data: $\delta\Psi$ (red, squares); $\delta\Delta$ (blue, triangles); $\lambda = 550$ nm; best-match model calculated TMO thickness parameter t_x (blue, triangles); best-match model calculated surface layer void fraction parameter f_{Void} (red, squares)]. Overlaid are the ALD deposition phases, indicated by roman numerals (See also Tabs. S1 and S2). Light-gray areas indicate phases without precursors present. Dark-gray areas to the left and right indicate neighboring cycles. Horizontal lines indicate the thickness GPC.
